# Supplementary material for: Learning curve and surgical outcome of robotic assisted colorectal surgery with ERAS program
Source: Sci Rep. 2022 Nov 29;12:20566. doi: 10.1038/s41598-022-24665-w (PMC9709162; doi:10.1038/s41598-022-24665-w)
Supplement: Supplementary file 2 — Supplementary Information 2. [file 41598_2022_24665_MOESM2_ESM.zip › RAL Raw data-Ñ[▒K/070/HS_ñw╝╨░O▒Kñσ.pdf]

正式摘要(電子簽章：00000213867158)<br>W92 / W92 ☐英文標題(注意:無英文姓名-請補病人基本檔!)	☐【讀取】憑證	

臺中榮民總醫院
出院病歷摘要

轉入號 : 轉出號 :

[redacted]

(8)流水編號	
(9)入院日期	110/03/31 (2021) 直腸外科 W92 - 33
(10)轉科日期	
(11)出院日期	110/04/04 (2021) 住院天數計 4 日

診斷

- 入院 : 1. Rectum, 10 cm from anal verge, Adenocarcinoma, moderately differentiated, arising in tubulovillous adenoma.
- 出院 : 1. Rectum, 10 cm from anal verge, Adenocarcinoma, moderately differentiated, arising in tubulovillous adenoma.
  - s/p robotic AR on 2021/04/01

主訴

- bloody stool for years

病史

- This 54 y/o male with a hx of
  1. DM not under control, 2021/03/31 HbA1c 9.2
  2. Hemorrhoid under conservative treatment
  FM: mother: colon cancer
  This time, he underwent colonscopy during health examination. Colonscopy showed multiple tumors at ascending colon and 40cm FAV, pathology showed tubular adenoma. However, rectal tumor at 10cm FAV was also noted and showed adenocarcinoma, moderately differentiated. Abdominal CT showed no obvious enlarged lymph nodes are noted over retroperitonium, masentary, and bilateral inginal fossa nor bony lesions. Under the impression of rectal cancer, adenocarcinoma, he was admitted for robotic LAR with ERAS protocol.

體檢發現

-
  - General appearance :
    - easy looking , conscious clear , without cardiopulmonary distress
    - Vital signs自動帶入date 體溫(BT) 脈搏(P) 呼吸(R) 血壓(BP) 身高(BH) 體重(BW) BMI
      20210331 36.7℃ 97次/分 17次/分 139/96mmHg 169.2cm 69.3kg 24.2
  - HEENT :
    - Eyes: no anemic conjunctiva, no icteric sclera, normal EOM
    - ENT: no otorrhea, no rhinorrhea, no erythematous mucosa, no injected tonsil, no deformity
  - Neck: supple, freely movable, trachea midline,no deformity, no engorged jugular vein , no palpable thyroid or lymph node enlargement
  - Chest:
    - Inspection: Grossly normal, symmetric and fully expansion, no accessory muscle used, no operation scar, no spider angiomata, no gynecomastia
    - Palpation: no palpable mass or nodule, no subcutaneous emphysema, no tenderness
    - Ausculation: clear breathing sound
  - Heart:
    - Percussion: normal heart size
    - Ausculation: regular heart beat, normal S1, S2, no gallop, no murmur
  - Abdomen:
    - Inspection: in

- - flat contour, symetrically, no scar, no stria, no dilated veins, no rashes
  - Ausculation: normoactive bowel sound, no abdominal bruits
  - palpation: soft, no muscle guarding or rigidity, no local tenderness, no rebounding pain, no palpable mass, no palpable pulsation
  - Percussion: no shifting dulllness, no hepatomegaly, no splenomagaly
-

Peripheral pulsation :	CA	BA	RA	FA	PA	PTA	DPA
Right side :	++	++	++	++	++	++	++/+++
Left side :	++	++	++	++	++	++	++/+++

- Genito-anus: no tenderness or palpable mass lesion or rectal shelf, normal external genitalia, normal anal tone, brown stool

CRS CONDITION

- - Lung

手術日期及方法

- 手術日期及方法20210401 Robot Assisted Low Anterior Resection
- 手術結果及發現：
  - 1. ulcerative tumor, around 1 cm in size at 6 cm FAV.

住院治療經過

- After admission, pre-op survey was done and there was no contraindication for operation. Robotic AR was perofrmed on 2021/04/01 and he tolerated the procedure. After op, he recovered well and no post-op complication was noted. He was discharged under the stable condition and would be followed-up at OPD.

合併症

- nil

一般檢查

- 累積報告：

DATE	TIME	ALB	TP	BIL.T	BIL.D	ALKP	AST	ALT	LDH	CHOL	TG	HDL-C	LDL	UA	NH3	AMY
1100402	0905			0.76	0.16		20	14								

DATE	TIME	WBC	RBC	HGB	HCT	MCV	PLT	BLST	NEUT	BAND	SEG	LYM	MON	EOS	BAS
1100402	0904	14900		13.9			234		85.2			8.5	6.1	0.0	0.2

DATE	TIME	NA	K	CL	CA	BUN	CREAT	Mg	P	CRP	ESR	PCT	LIPASE	LACTATE
1100402	0905	138	4.2		8.6	10	1.06							

特殊檢查

病理報告

- PENDING

其它

出院時情況

- 3.改門診治療

出院指示

藥名	劑量	劑量單位	用法	頻次	數量	數量單位	現況	天數	單位
Ultracet tab	1	TAB	PO	Q6HPRN	40	TAB	TH	10	天
Flurbiprofen 100mg	1	TAB	PO	BID	20	TAB	TH	10	天
Mag. Oxide tab	2	TAB	PO	TID	60	TAB	TH	10	天

出院計劃

主治醫師 2062F 蔣鋒帆 / 住院醫師 2334J 陳勇先

正式摘要(電子簽章：**000000213867158)**

W92 / W92

doctorRank:B
